# Gpu-accelerated JEMRIS for extensive MRI simulations

**DOI:** 10.1007/s10334-025-01281-z

**Published:** 2025-09-04

**Authors:** Aizada Nurdinova, Stefan Ruschke, Michael Gestrich, Jonathan Stelter, Dimitrios C. Karampinos

**Affiliations:** 1 Department of Radiology, Stanford University, Stanford, USA; 2 Institute of Diagnostic and Interventional Radiology, School of Medicine and Health, Technical University of Munich, Munich, Germany; 3 Altair Engineering GmbH, Boeblingen, Germany

**Keywords:** GPU acceleration, JEMRIS, Bloch simulations, Motion artifacts, Quantitative MRI

## Abstract

**Purpose:**

To enable accelerated Bloch simulations by enhancing the open-source multi-purpose MRI simulation tool JEMRIS with graphic processing units (GPU) parallelization.

**Methods:**

A GPU-compatible version of JEMRIS was built by shifting the computationally expensive parallelizable processes to the GPU to benefit from heterogeneous computing and by adding asynchronous communication and mixed precision support. With key classes reimplemented in CUDA C++, the developed GPU-JEMRIS framework was tested on simulations of common MRI artifacts in numerical phantoms. The accuracy and performance of the GPU-parallelized JEMRIS simulator were benchmarked against the CPU-parallelized JEMRIS and GPU-enabled KomaMRI.jl simulators. Additionally, an example of liver fat quantification errors due to respiratory motion artifacts was simulated in a multi-echo gradient echo (MEGRE) acquisition.

**Results:**

The GPU-accelerated JEMRIS achieved speed-up factors 3–12 and 7–65 using double and single precision numerical integrators, respectively, when compared to the parallelized CPU implementation in the investigated numerical phantom scenarios. While double precision GPU simulations negligibly differ (<0.1% NRMSE) from double precision CPU simulations, the single precision simulations still present small errors of up to 1% k-space signal NRMSE. The developed a GPU extension enabled computationally demanding motion simulations with a multi-species abdominal phantom and a MEGRE sequence, showing significant and spatially varying fat fraction bias in the presence of motion.

**Conclusion:**

By solving the Bloch equations in parallel on device, accelerated Bloch simulations can be performed on any GPU-equipped device with CUDA support using the developed GPU-JEMRIS. This would enable further insights into more realistic large spin pool MR simulations such as experiments with large multi-dimensional phantoms, multiple chemical species and dynamic effects.

**Supplementary Information:**

The online version contains supplementary material available at 10.1007/s10334-025-01281-z.

## Introduction

Nuclear magnetic resonance (NMR) concepts are governed by the laws of quantum mechanics. However, using macroscopic phenomenological equations of nuclear magnetization motion, specifically the Bloch equations [1], MR imaging phenomena can be accurately described based on a concise classical formulation. Therefore, MRI simulations based on solving Bloch equations (Bloch simulations) present a straightforward, precise and powerful tool in MRI research and education [2, 3]. Although scrupulous, they can include most magnetization phenomena during MR data acquisition, and, therefore, provide valuable insights into understanding MRI effects compared to simplified methods, such as using Fourier transform-based model assumptions. Furthermore, Bloch simulations can be adapted to naturally include dynamic effects, including motion or diffusion. Bloch simulation-based approaches can be utilized in pulse sequence design and optimization [4, 5], ground truth data generation and image synthesis [6], quantitative imaging [7] and the study of artifacts and hardware imperfection [8, 9].

MR imaging problems often involve complex multi-tissue objects, advanced sequence designs with complex gradient waveforms, multi-coil transmission and reception, and dynamic effects (e.g. motion or dynamically varying contrast). Therefore, performing realistic Bloch simulations can be challenging and require significant computational resources. There have been numerous efforts in overcoming the Bloch simulations computational burden by developing software solutions that can be used on a single or multiple GPUs. Many of the previously proposed Bloch simulators are proprietary [10–13], while a bunch of recent frameworks are open-source [14–17]. Among these, KomaMRI.jl [14] stands out for its user-friendly design, efficient and flexible simulation capabilities. Another well-established GPU simulators are MRiLab, which supports multi-pool exchange modeling, as well as MR-STAT [15] and MATI [16] frameworks particularly well-suited for tissue parameter mapping applications.

Bloch simulations can be performed using either numerical differential equation solvers [18] or a matrix formulation [19]. Each approach has its strengths and weaknesses, depending on the specific problem being studied. Using numerical ordinary differential equation (ODE) solvers offers a brute-force general approach that can incorporate all relevant effects simultaneously, including precession, relaxation, and other dynamics. These solvers can adapt time-stepping depending on problem complexity and provide high accuracy, even for rapidly varying fields. However, they can be computationally expensive, especially when small time steps are required for solution stability. In contrast, Bloch matrix simulations are often computationally more efficient and naturally suited for parallelization. However, they require careful dissection of the MR sequence to properly set time steps, and often rely on assumptions such as simplified operators or splitting precession and relaxation effects. Additionally, their accuracy may be theoretically limited for fast-changing fields since they approximate dynamics by dividing the field into piecewise-constant segments. Most of the mentioned open-source Bloch simulators are based on analytical matrix solutions with small timesteps [14–17].

The open-source JEMRIS framework [18] directly solves the Bloch ODEs with high precision, it has been widely adopted by researchers due to its efficient adaptive time step ODE solvers, general and intuitive pulse sequence structure as well as the availability of Graphical User Interface (GUI) and CPU parallelization. Over the years, JEMRIS has been further advanced by the community to support Pulseq [20], flow and complex motion effects [21, 22], multi-pool spin systems [23] and ISMRMRD format [24]. Recently, it was also included in the comprehensive imaging workflow for reproducible MR studies [24]. However, JEMRIS remains up to now not compatible with GPU parallelization and heavily computationally demanding while studying problems with a large pool spin systems and dynamic effects. Nevertheless, GPU acceleration would be now feasible, thanks to the development of GPU-aware ODE solvers [25], which form the computational core of JEMRIS.

The present work aims to develop an extension for the open-source JEMRIS software using a CUDA-enabled GPU processing (GPU-JEMRIS). While numerous GPU-accelerated simulators are available as open-source tools, the present work constitutes an alternative approach, as JEMRIS remains a general and extensive framework with minimal assumptions when solving the Bloch equations using variable-step ODE solvers. The developed GPU-JEMRIS adds up to the software convenience by allowing powerful simulations to be performed on any computer without much CPU power, but equipped with GPU hardware. The present manuscript describes the GPU-JEMRIS implementation, shows performance benchmarks and presents a dynamic simulation case by simulating liver proton density fat fraction (PDFF) quantification errors induced by respiratory motion effects.

## Background

### Bloch simulations

Bloch simulations are based on the phenomenological equations [1] describing the dynamics of the magnetization vector $\vec {M}$ in the presence of the magnetic field $\vec {B}(t)$. In the rotating frame of reference at the resonance frequency $\omega _0$, the equations take the form:

1 $$\begin{aligned} \frac{\partial \vec {M}}{\partial t} = \vec {M}(t)\times \gamma \vec {B}_{eff} - \frac{M_{x}\vec {i}+M_{y}\vec {j}}{T_2} - \frac{(M_{z}-M_z^0)\vec {k}}{T_1}, \end{aligned}$$

2 $$\begin{aligned} \vec {M}(0) = [\;0, 0, M_z^{0}\;]^{T},\; \gamma \vec {B}_{eff} = [\gamma B_{1x}, \gamma B_{1y},\Delta {\omega _{RF}}], \end{aligned}$$

where $\Delta {\omega _{RF}} = \omega - \omega _0$ is defined by total additional fields due to spatial encoding and off-resonance effects, $\vec {i}$, $\vec {j}$ and $\vec {k}$ are coordinate unit vectors, $M_z^0$ is the thermal equilibrium magnetization, $T_1$ and $T_2$ are time constants characterizing relaxation processes, $B_1$ is the radiofrequency (RF) field, while $B_0$ is the main field along *z*-axis. The constant $\gamma$, the gyromagnetic ratio, is the ratio of the angular momentum of a particle to its magnetic moment. The system can be written in a linear form to notice a few properties:

3 $$\begin{aligned} \dot{\vec {M}}= & \underbrace{ \begin{pmatrix} -1/T_2 & \Delta \omega _{RF} & -\gamma B_{1y} \\ -\Delta \omega _{RF} & -1/T_2 & \gamma B_{1x} \\ \gamma B_{1y} & -\gamma B_{1x} & -1/T_1 \end{pmatrix}}_{A} \cdot \vec {M} + \underbrace{ \begin{pmatrix} 0 \\ 0 \\ M_z^0/T_1 \end{pmatrix}}_{b} = A\; \vec {M} + b = f(t, \vec {M}), \end{aligned}$$

where $\dot{\vec {M}} = \frac{\partial M}{\partial t}$, *A* is spatiotemporal coefficient matrix.

Generally, the system Eq. 3 takes the coupled ODE system form due to the non-zero cross-product. Although the analytical solution might exist and could be found by decoupling the system as in [26], numerical methods are more practical at solving this system avoiding the need for analyzing each timepoint in the sequence. The system matrix in Eq. 3 is a sum of a skew-symmetric and diagonal matrices, and has distinct negative real part eigenvalues. Therefore, the system is stable and appears to have no large stiffness values with realistic tissue parameters [26]. For this reason, simple explicit linear methods can be used for solving Eq. 3.

Alternatively, one can choose implicit methods, which unconditionally improve the stability of the solution even for stiff ODEs. For either choice of the method, variable-order, variable-step multistep approaches, such as in the CVODE package [25, 27] allow to automatically maintain the desired accuracy while reducing computation by adjusting the step size to the dynamics.

### JEMRIS formulation and solver

The open-source simulator JEMRIS was previously developed in C++ with code encapsulation allowing for a convenient project customization and extension [18]. MRI simulations using JEMRIS are suitable for any numerical phantom and any sequence, incl. from clinical scanners, converted into an.*xml* file format. Moreover, effects during the MR encoding, such as relaxation, motion, diffusion, flow, etc., can be precisely included in the framework. JEMRIS [18] formulates the Bloch equations Eq. 1 in cylindrical coordinates in the rotating frame of reference:

4 $$\begin{aligned} & \frac{\textrm{d}}{\textrm{d}t}\begin{pmatrix} M_r\\ \phi \\ M_z \end{pmatrix} = \begin{pmatrix} \cos \phi & \sin \phi & 0\\ -\frac{\sin \phi }{M_r} & \frac{\cos \phi }{M_r} & 0 \\ 0 & 0 & 1 \end{pmatrix}\nonumber \\ & \quad \cdot \begin{bmatrix} \begin{pmatrix} -1/T_2 & \Delta \omega _{RF} & -\gamma B_{1y} \\ -\Delta \omega _{RF} & -1/T_2 & \gamma B_{1x} \\ \gamma B_{1y} & -\gamma B_{1x} & -1/T_1 \end{pmatrix} \cdot \begin{pmatrix} M_r\cos \phi \\ M_r\sin \phi \\ M_z \end{pmatrix} + \begin{pmatrix} 0 \\ 0 \\ M_0/T_1 \end{pmatrix} \end{bmatrix} \end{aligned}$$

The simulator uses implicit Adams–Moulton solvers from the CVODE package [27]. The method requires an implicit multistep approach where nonlinear terms must be handled using iterative solvers such as Newton’s method, fixed-point iteration (used in v2.8), or diagonal approximation (used in v2.9). The numerical ODE integrator updates magnetization iteratively at each time step $t_n \rightarrow t_{n+1}$ using the nonlinear equation:

5 $$\begin{aligned} y_{n+1} = y_n + h \beta f(t_n, y_{n+1}), \end{aligned}$$

where $y_n$ is the solution vector equal to magnetization $\vec {M}$ from Eq. 1, at time $t_n$, and *h* is the integration step size and $f(t_n, y_{n+1})$ is the RHS function. Since $y_{n+1}$ appears implicitly, it must be iteratively refined using a fixed-point approach:

6 $$\begin{aligned} y_{n+1, i}^{(m+1)} = y_n + h \beta f(t_n, y_{n+1, i}^{(m)}), \end{aligned}$$

where *i* indexes each element of the solution vector, and *m* denotes the iteration count within the solver. The process continues until convergence determined by the maximum element-wise difference between successive iterations:

7 $$\begin{aligned} \max _i |y_{n+1, i}^{(m+1)} - y_{n+1, i}^{(m)}| < \text {tolerance}. \end{aligned}$$

The computational burden from solving the ODE system in JEMRIS is overcome with parallel computations using the Message Passing Interface (MPI) framework.

### GPU acceleration in Bloch simulations

For Bloch simulations, an ensemble of spins with magnetic properties ${\textbf{P}}({\textbf{r}}, t)$ is simulated with Eq. 4 in the presence of a total magnetic field ${\textbf{B}}({\textbf{r}}, t)$:

8 $$\begin{aligned} {\textbf{P}}({\textbf{r}},t) = & [\mathbf {M_0}({\textbf{r}},t),\; \mathbf {T_1}({\textbf{r}},t),\; \mathbf {T_2}({\textbf{r}},t),\; \mathbf {T_2^*}({\textbf{r}},t),\; \nonumber \\ & \quad \varvec{\Delta }\omega _{\textbf{RF}}({\textbf{r}},t),\; {\chi }({\textbf{r}},t)], \; {\textbf{B}} = {\textbf{B}}({\textbf{r}}, t) \end{aligned}$$

For GPU-parallelized implementation, numerical ODE integrator can operate on device for all spins simultaneously iterating over timepoints *t*:

9 $$\begin{aligned} \dot{\tilde{{\textbf{M}}}}(t) = f(t,\tilde{{\textbf{M}}}) = J\,{\textbf{A}}(t) \tilde{{\textbf{M}}} + {\textbf{b}},\;\; \tilde{{\textbf{M}}}(t_0) = \mathbf {M_0}, \end{aligned}$$

where *N* is the number of simulated spins, $\tilde{{\textbf{M}}} \in R^{\,3N\times 1}$ and $\dot{\tilde{{\textbf{M}}}}(t) = \partial \tilde{{\textbf{M}}}/\partial t$ are concatenated magnetization and its derivative vectors for all spins in the cylindrical coordinate system, *J* is the coordinate change matrix. The right-hand-side (RHS) function $f: R^{\,3N\times 1} \rightarrow R^{\,3N\times 1}$, ${\textbf{A}} \in R^{\,3N\times \,3N}$ and ${\textbf{b}} \in R^{\,3N\times 1}$ are defined as in Eq. 3 but for all spins based on ${\textbf{P}}({\textbf{r}},t)$ and ${\textbf{B}}({\textbf{r}},t)$.

The iterative scheme defined by Eqs. 5, 6, 7 is applied now to vector $y = \tilde{{\textbf{M}}}(t)$ containing magnetizations for all spins. It can be efficiently parallelized on GPU, where each element of *y* is assigned to a separate thread, performing independent updates. The right-hand-side function *f* is evaluated using kernel function, followed by convergence checking via a parallel reduction on device. This enables solving large ODE systems efficiently by leveraging coalesced memory access and device-wide synchronization.

Moving computationally demanding tasks to the GPU helps accelerate Bloch simulations, but introduces overheads due to device memory allocation and data transfers between the CPU and GPU. These overheads can be reduced by using the concurrency principle in the CUDA programming model, where host (CPU) and device (GPU) computations, as well as data transfers, are treated as independent tasks that can run simultaneously. Concurrency can be achieved in various ways, such as using pinned host memory and launching kernels on non-default CUDA streams to allow asynchronous data transfers and computations. Additionally, GPU-based Bloch simulations involving iterative numerical calculations can significantly improve performance and resource efficiency by optimizing for lower precision (e.g., single or half precision). For instance, performance ratios for single precision (FP32) compared to double precision (FP64) are reported to range from approximately 1:2 for professional-grade GPUs to 1:20 for consumer-grade GPUs. However, lower precision may affect the accuracy and stability of the calculations. Therefore, it is important to balance performance optimization with the precision requirements of the specific application to ensure reliable results.

## Methods

### GPU-JEMRIS: main implementation

**Fig. 1:**
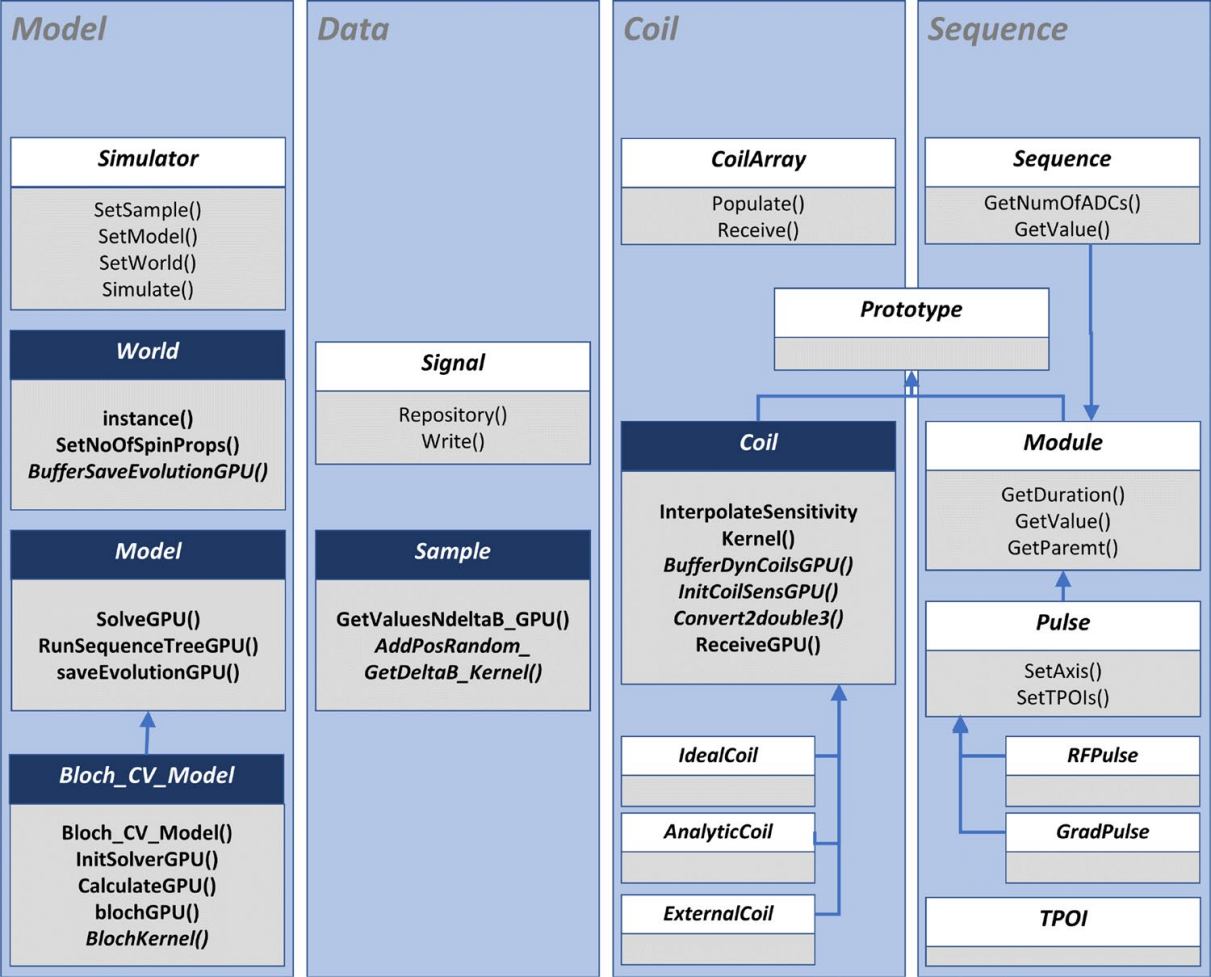
Schematic class diagram of GPU-JEMRIS highlighting main changes for the GPU support in dark blue boxes. The following functionalities are modified: methods allocating GPU memory and copying data of the computational core to device are added to classes *World*, *Sample* and *Coil*. The class *Model* communicates with the sequence tree on the host and calls the solver on the GPU at the timepoints of interest (TPOI) in *RunSequenceTreeGPU()*. The numerical solver on GPU is implemented in *Bloch_CV_Model*: the function *blochGPU()* computes the ODEs right-hand side for all spins in parallel eliminating the sequential loop in the original CPU version. The *Coil* class interpolates input sensitivity maps and keeps them on device throughout the simulations. The Bloch equations are then solved for all spins at every TPOI, the *Coil* method *ReceiveGPU* performs asynchronous reduction on device. The *Sequence* class is not modified for the GPU support

The JEMRIS source code can be, in general, clustered into functionalities handling model, input/output data, coil and sequence. To add CPU-GPU heterogeneous computing in CUDA C++, the JEMRIS classes *Model*, *Bloch_CV_Model*, *World*, *Sample* and *Coil* were re-implemented. The changes are summarized in Fig. 1 and described in detail below. For GPU-accelerated ODE solving, the necessary steps include compiling the library with GPU support, utilizing device-enabled N_Vector objects, verifying GPU compatibility of (non)linear solvers, and defining the right-hand side (RHS) along with other required kernels. The solver control remains on the host, so no modifications were made to the recursion through the JEMRIS sequence tree. Host-device data migration is managed externally from CVode [25].

First, as GPU-JEMRIS operations are performed for all spins simultaneously, the class *Sample* is modified to allocate arrays containing tissue properties for the whole ensemble, ${\textbf{P}}({\textbf{r}},t)$ in Eq. 4. The allocated vectors are transferred to the device, where random variables for simulating effects such as off-resonance and $T_2^{*}$ are generated directly on the GPU. Since the data transfer and random variable generation are independent for all spins, these operations are executed asynchronously using multiple CUDA streams. To achieve this, the *PinEnsembleGPU* method handles data type conversion and CPU memory pinning. This is followed by asynchronous data transfer to device and randomized position and off-resonance generation using kernels using the *cuRAND* library. Once the sample is fully prepared on the device, the coil sensitivity maps $C({\textbf{r}})$ are initialized, transferred to GPU, and interpolated on device.

Second, the classes *World*, *Model* and *Bloch_CV_Model* are modified to initialize the GPU solver and the *CUDA N_Vector* arrays $\tilde{{\textbf{M}}}({\textbf{r}},t)$ and $\dot{\tilde{{\textbf{M}}}}({\textbf{r}},t)$ for magnetization and its derivatives for all spins. As the *Sequence* class is not changed for GPU-JEMRIS, the simulator still runs through the sequence tree and computes both the total magnetic field ${\textbf{B}}({\textbf{r}},t)$ and the next timepoint of interest (TPOI) on the host. However, the ODE solver is invoked on the device for the whole ensemble at every TPOI. In this way, the adaptive stepping feature is preserved for the numerical solver. *BlochKernel* defines the ODE right-hand-side function to solve the system Eq. 9 with the Adams–Moulton method and fixed-point (functional) iterations using CUDA functionalitites of the SUNDIALS library [27].

Third, the signal receive operation is implemented asynchronously for multiple coil channels as a reduction operation using the optimized *CUB* CUDA library. Block size for kernel launches was empirically defined as 512 in *World*.

**Fig. 2:**
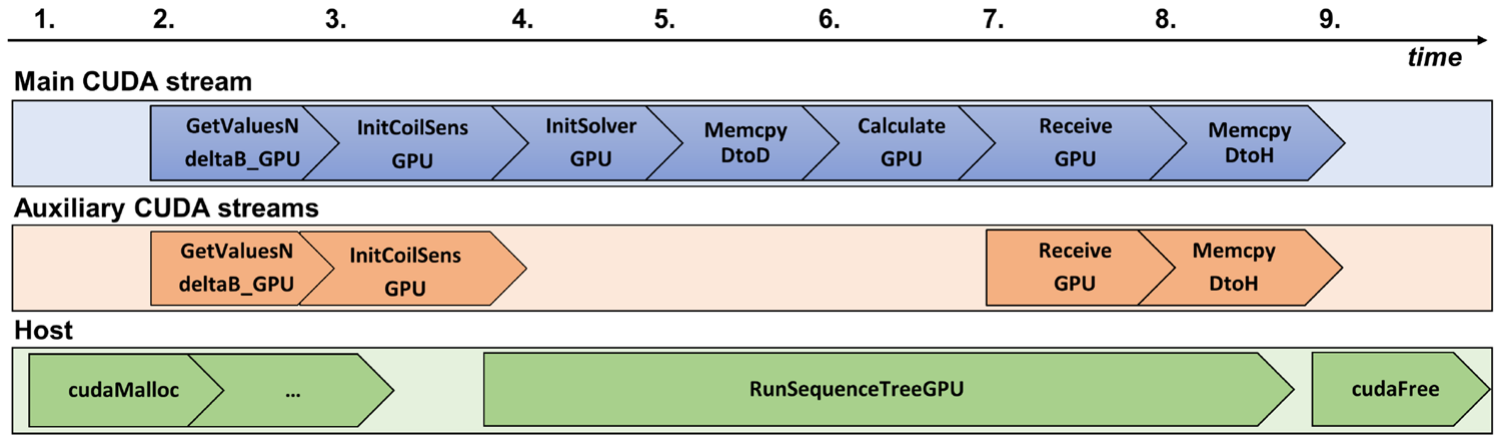
The train of function calls during GPU-JEMRIS simulations in the main and helper GPU streams, as well as in the CPU thread. Note that after GPU memory allocation, the host and CUDA streams run independently. Steps 1–3 involve data preparation, GPU memory allocation, and data transfer to the device (GPU). Steps 4–6 include iteration through the sequence tree on the host, CVODE model re-initialization, and solving Bloch equations on the device for each time point of interest (TPOI). At step 7, the estimated magnetization is multiplied by coil sensitivities and summed on the GPU. Steps 8–9 involve transferring the results from device to CPU, freeing up resources and completing the simulation process

Figure 2 shows the relative timeline of functions on host and device. In the simulation start-up stage, steps 1–3, all mentioned GPU arrays are allocated and transferred from CPU. Step 4 includes the numerical ODE solver initialization on device and non-default CUDA streams allocation in the *Bloch_CV_Model* constructor, as well as $\tilde{{\textbf{M}}}({\textbf{r}},t)$ and $\dot{\tilde{{\textbf{M}}}}({\textbf{r}},t)$ initalization in *InitSolverGPU* of the *Bloch_CV_Model*. Further, in the ODE-solving stage, steps 5–6, the method *RunSequenceTreeGPU* in the class *Model* communicates with the sequence, re-initializes and calls the solver on GPU at TPOIs. For this, the *Bloch_CV_Model* methods *InitSolverGPU* and *CalculateGPU* are invoked. Dynamic quantities, such as sample parameter maps, spin positions and total field values are updated on device at every TPOI. Step 7 receives the calculated multi-coil magnetization at every readout timepoint, while step 8 transfers the total magnetization vector from device to host. After the time-loop in *RunSequenceTreeGPU* is finished, all device memory is freed at step 9. The timing results presented in this paper will include all steps 1–9.

### Optimization of GPU-JEMRIS features

The GPU extension for JEMRIS was further optimized by adopting the following three optimization steps: (a) memory initialization only during the simulation start-up, (b) an asynchronous and minimized number of host-to-device and device-to-host data transfers, as well as asynchronous kernel launches, and (c) an optional single precision arithmetic.

Device memory allocation is a synchronous operation, which means the host cannot launch other commands while the GPU is busy with the allocation. In addition, every call of the allocation function has significant overhead. Therefore, all memory initializations in GPU-JEMRIS were gathered in the numerical model preparation stage (steps 1–3 in Fig. 2) and were avoided inside loops (such as steps 4–8).

Asynchronous data transfers and kernel computations were implemented using non-default CUDA streams and pinned memory. Five CUDA streams were allocated during the *Model* class object construction: the first stream runs most of the operations, and the other four streams support by handling computations not requiring results from the main stream (Fig. 2). For example, data transfers can run in parallel with the model initialization for the next time step, or multi-coil reception can be performed for all channels independently, so these operations can be split between the main and auxiliary streams. Each memory transfer and kernel start have an execution overhead, so it is beneficial to call these fewer times on larger chunks of data, which was accounted for by merging some arrays on the GPU.

As mentioned before, many GPU devices exhibit limited double-precision arithmetic performance compared to single precision computations, therefore, GPU-JEMRIS was implemented to allow operating on the *float* data type. For this, the CVode numerical integrators were compiled with single precision and device data necessary to formulate the Bloch equations as described in the previous section was converted from double to single precision. Although such explicit conversion is not efficient, the performance gain due to GPU single precision computations was confirmed to be much higher for the investigated setup. However, the rounding errors accumulate much faster in single precision compared to double precision when summing millions of spins during the signal reception process. Therefore, GPU-JEMRIS performs multiplication to receive coil sensitivity maps and transverse magnetization summation in double precision for both the double and single precision GPU models.

**Fig. 3:**
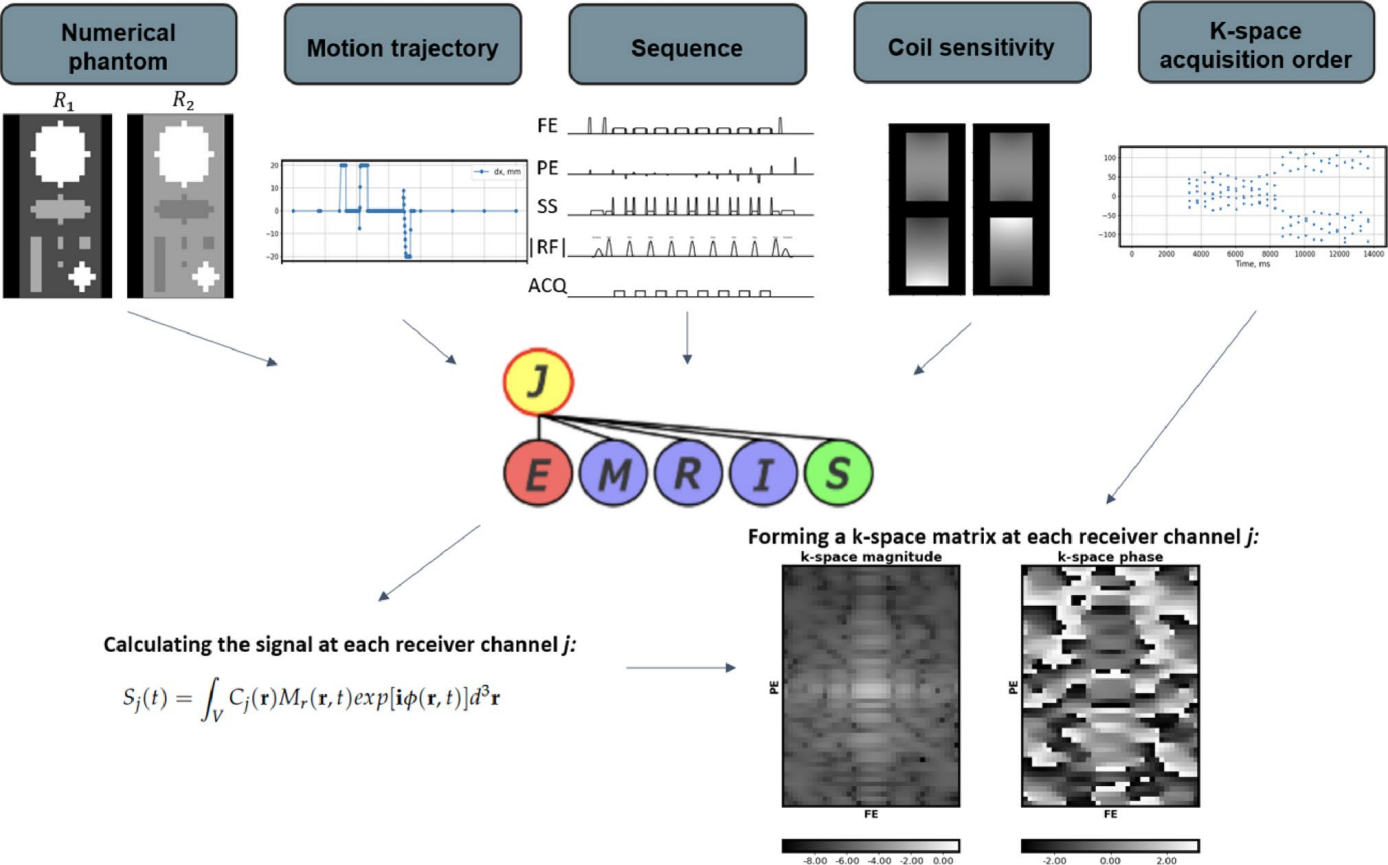
JEMRIS simulations of motion artifacts: input parameters are a numerical phantom with set magnetic properties ($T_1$, $T_2$, $T_2^*$, *PD*, $\Delta \omega$, $\chi$), a motion trajectory as a function of time, an acquisition sequence, transmit/receive coil sensitivity maps. The output of the simulator is the bulk magnetization vector at the acquisition timepoints, which can be converted to k-space matrix by taking the transverse magnetization part, provided the phase encoding order in time

### Experiments

The Bloch simulation pipeline using JEMRIS is illustrated in Fig. 3. The expected inputs include a numerical phantom as in Eq. 8, dynamic parameter trajectories such as motion, MR sequence, and transmit and receive coil sensitivity maps. The simulator outputs the multi-channel received magnetization at the ADC points, which can be converted into a k-space matrix, provided the k-space acquisition ordering.

#### Simulation accuracy

The accuracy of the developed GPU-JEMRIS was evaluated based on three simulations of MRI artifacts from [18], also included in the example sets of the JEMRIS website [28]. First, the chemical shift artifact was simulated in an echo planar imaging (EPI) acquisition, based on a numerical brain phantom containing fat tissue and assuming uniform transmission and reception. Second, banding artifacts were simulated in a balanced steady-state free precession (bSSFP) acquisition, based on a numerical brain phantom including susceptibility values for air in the sinuses region and assuming uniform transmission and reception. Third, the artifact due to a long refocusing pulse (duration of the order to $\sim T_2$) was simulated in a spin-echo acquisition, based on a numerical brain phantom with susceptibility and chemical shift effects and assuming uniform transmission and reception. For all three artifact examples, 140,000 spins were simulated, and signals as well as computation times were compared between double and single precision GPU-JEMRIS simulations and CPU-JEMRIS simulations parallelized with MPI.

#### Simulation speed

**Fig. 4:**
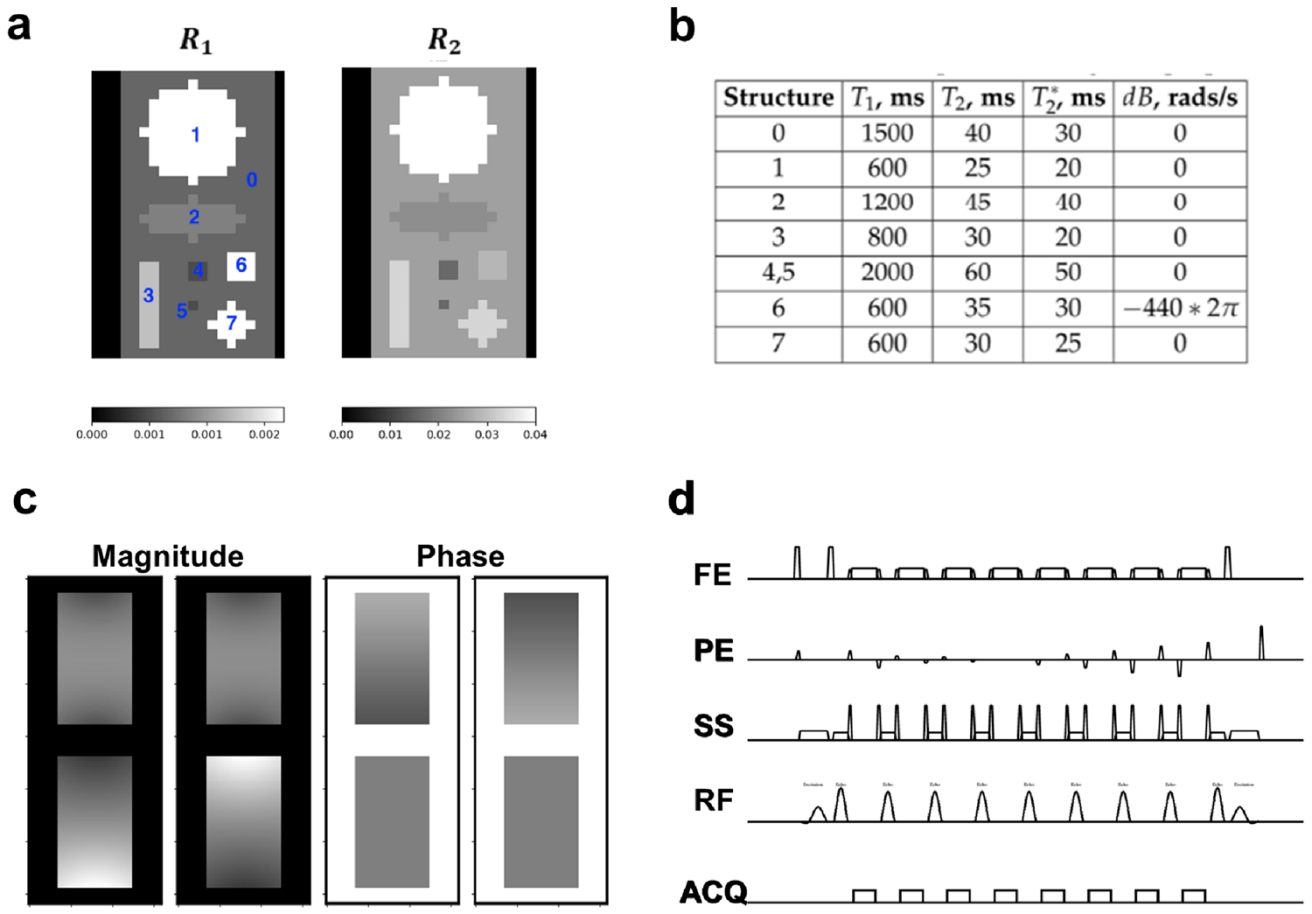
Geometrical phantom simulations setup: (**a**) $R_1 = 1 / T_1$ and $R_2 = 1 / T_2$ maps (units [1/s]) of the constructed numerical phantom containing geometrical shapes enumerated 1–7 with MR properties as depicted in table (**b**). (**c**) Magnitude and phase maps of the simulated 4-channel reception coil array. (**d**) Simulated 2D multi-shot TSE sequence exported from the clinical scanner, sequence parameters are provided in Table 1

The speed of the developed GPU-JEMRIS was evaluated in simulations with a variable number of spins: $[2250,\; 22,500,\; 225,000,\; 2,250,000]$. Specifically, a numerical phantom including geometrical structures with assigned MR properties (Fig. 4a, b) was simulated with a 2D turbo spin echo (TSE) sequence described below. A 4-channel birdcage receiver coil array was simulated using Sigpy v0.1.23 (Fig. 4c) [29]. Signals and computation times were compared between double and single precision GPU-JEMRIS simulations and MPI-CPU-JEMRIS simulations.

Further, the simulation speed of GPU-JEMRIS was compared to KomaMRI.jl in five gradient echo (GRE) simulations presented in [14]: (a) *EPI of a 1D Column* of spins, divided into four segments with properties $M_0 = [1,\; 0.5,\; 1, 0.5]$ and $T_1 = T_2 = [100,\; 50,\; 100,\; 50]$ ms. (b) *EPI of Concentric Circles* with radii $R = 50$ mm and $r = 25$ mm, where the inner circle had a frequency offset $\Delta \omega = 200$ rad/s. Both had $M_0 = 1$, but different relaxation times: $T_1 = T_2 = 50$ ms (inner) and 100 ms (outer). (c) *EPI of a Brain Phantom* incorporating a realistic off-resonance field with $\Delta \omega$ from $-400$ to 1200 rad/s. (d) *EPI of the Brain Phantom with Motion*, applying displacement $u_y(x, t) = v_y t$ with $v_y = 0.1$ m/s, without off-resonance. (e) *Spiral GRE of the Brain Phantom* without off-resonance, with echo time $TE = 0.1$ ms. All experiments used GRE sequences with hard RF pulses, a field of view (FOV) of $230 \times 230$ $\hbox {mm}^2$, spatial resolution $\Delta x = 2.3$ mm, phantom resolution $\Delta x_{\text {obj}} = 1$ mm, and uniform reception. The KomaMRI.jl simulations were timed 10 times in Julia after a warm-up run for just-in-time (JIT) compilation, whereas JEMRIS simulations were timed 10 times from the command line.

#### Simulation of quantitative imaging in the presence of motion

An example of quantitative imaging in the presence of motion was studied to evaluate the developed GPU-JEMRIS simulations with additional dynamic effects. Specifically, artifacts induced by respiratory-like motion were assessed in a chemical shift encoding-based water-fat separation measurement of the abdomen.

**Fig. 5:**
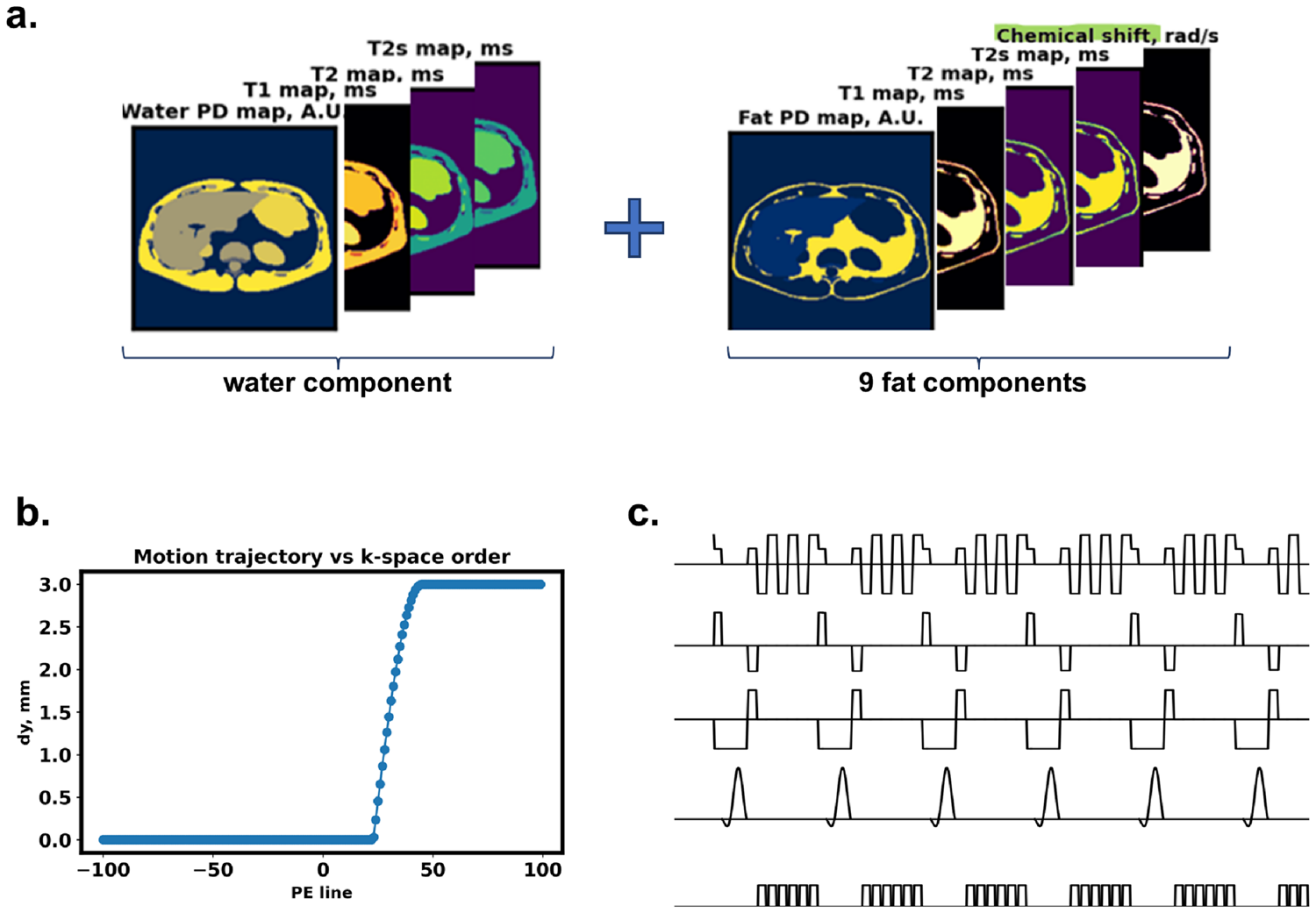
Liver phantom simulations setup. (**a**) DUKE abdominal numerical phantom with assigned magnetic properties: the phantom includes one water and nine fat components with chemical shifts and relative proton density (PD) values varying according to the Hamilton fat model [31]. (**b**) Translational motion as a function of acquired k-space phase encoding line. (**c**) Sequence diagram of the quantitative six-echo gradient echo sequence exported from the clinical scanner and converted to the .*xml* format suitable for JEMRIS. Sequence parameters are provided in Table 1

The Duke numerical phantom [30] was used to create a water-fat abdominal phantom by combining one water and nine fat species with assigned magnetic properties as shown in Fig. 5a. Proton density and chemical shift values for the fat species were set according to an in vivo spectroscopy-based fat model [31]. Figure 5c presents the sequence diagram of five repetition times (TR) of the multi-echo gradient echo (MEGRE) sequence used for the simulation. Handling the GRE sequence with short TR required tuning the number of spins per voxel for gradient spoiling. For this purpose, the GRE acquisition was additionally tested with a homogeneous disc phantom, and it was found that at least 100 spins per voxel need to be simulated (Suppl. Fig. S4). The simulated motion trajectory against the timing of the acquired phase-encoding lines is presented in Fig. 5b. Anterior-posterior translational motion by 3 pixels is assumed to occur in the second half of the data acquisition. Transmission and reception coil fields were kept uniform, and the simulations were performed on the GPU with single precision.

**Fig. 6:**
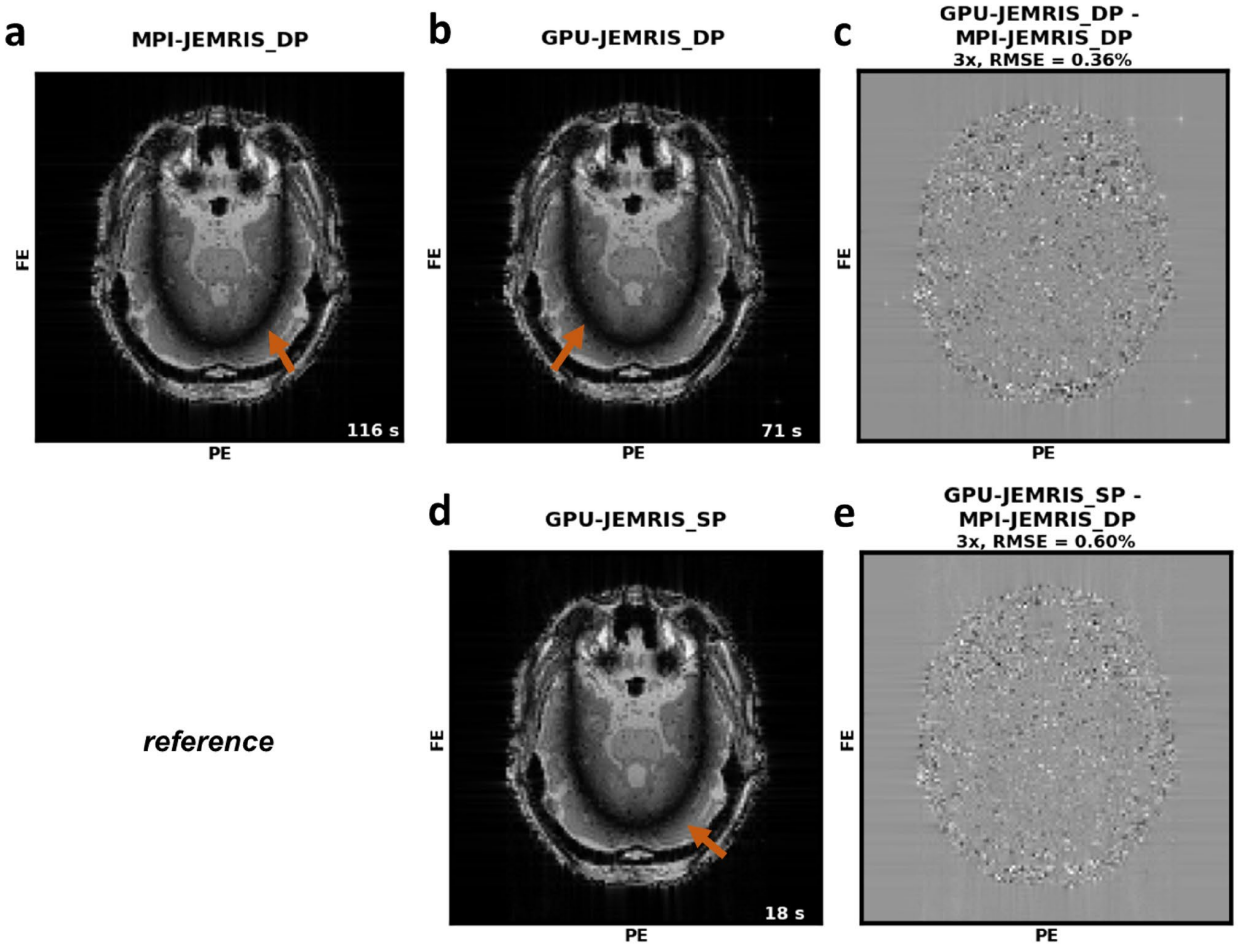
Accuracy of GPU simulations relative to CPU computations for bSSFP banding artifacts induced by susceptibility in a brain phantom (reproducing results from [18]). (**a**) The reconstructed image from the MPI simulations which took 116 s. (**b**) Image simulated using double precision GPU-JEMRIS, the computation took 71 s. (**c**) Image difference with NRMSE = 0.36% between the double precision MPI and double precision GPU computations. (**d**) Image simulated using single precision GPU-JEMRIS, the computation took 18 s. (**c**) Image difference with NRMSE = 0.6% between the double precision MPI and single precision GPU computations. Both figures (**c**) and (**e**) indicate noise-like differences between images from CPU-based and GPU-based simulations

Assuming non-interacting spins, the simulated magnetization signals from water and fat species were normalized by the number of spins and summed according to the model [31]. Received magnetization at the six echo times formed the k-space signals, which inverse Fourier transform images were fed to an implemented complex-based water-fat separation algorithm [32, 33].

#### MRI sequence waveforms and parameters

To keep the speed benchmarks and quantitative imaging simulations more realistic, a $T_1$-weighted multi-shot TSE sequence (for the speed simulation) and a $T_1$-weighted Dixon MEGRE sequence (for the quantitative imaging simulation) were exported from a clinical 3 T Elition scanner (Philips Healthcare, The Netherlands). The sequence parameters are indicated in Table 1 and the relevant sequence diagrams provided in Figs. 4d and 5c. The sequence files obtained from the scanner were converted into the *.xml* format compatible with JEMRIS using a specialized tool provided under Philips research agreement.

**Table 1:** Scan parameters of the $T_1$-weighted DRIVE turbo spin echo (TSE) and the six-echo multi-echo gradient echo (MEGRE) sequences exported from the 3 T Elition scanner (Philips Healthcare, The Netherlands)

Parameter	${T_1}$w-DRIVE TSE	6-echo MEGRE
TE$_1$/$\Delta$TE (ms)	18/–	3.1/1.3
TR (ms)	450	14
Flip angle ($^{\circ }$)	90	3
FOV (mm$^3$)	64 $\times$ 64 $\times$ 2.5	200 $\times$ 200 $\times$ 1
Voxel size (mm)	1	1
Oversampling	No	No
TSE factor and order	8, asymmetric	–
Echoes	1	6
Slice orientation	Sagittal	Transverse
Acceleration	CS-SENSE ($R = 2$)	No

#### Workstation specifications

The accuracy and speed simulations were carried out on a workstation with $2 \times \text {Intel Xeon Gold }6252$ ($24\times 2.1$ GHz) CPUs and $\text {NVIDIA Titan RTX }$ (24 GiB) GPU. The performance of a single CPU socket was compared to that of a single GPU device. Simulations of the liver phantom with the quantitative MEGRE sequence, as well as benchmarks against KomaMRI.jl were performed on a workstation with $\text {Intel Xeon W-2145 }$ ($8\times 3.7$ GHz) CPU and $3\text { NVIDIA RTX A6000}$ (48 GiB) GPUs.

#### Code availability

GPU-JEMRIS source code, Dockerfiles (for CPU-, MPI- and GPU-JEMRIS) encapsulating software dependencies, as well as the scripts for the described experiments are posted on GitHub under https://github.com/BMRRgroup/gpu-jemris.

## Results

### Optimization results

The simulation timelines generated by the Nsight profiler for a two spin-echo experiment with 2 million spins (Suppl. Fig. S1) indicate the efficient usage of the GPU device. In the GPU-JEMRIS simulations in Suppl. Fig. S1a, the device is being occupied for the whole simulation runtime and GPU memory allocation is mainly at the start. The zoomed-in views in Suppl. Fig. S1b-d indicate densely-packed tasks with overlaps when independent device memories were used for operations. Suppl. Fig. S1f shows the profile without dead times during solving the Bloch equations, which take up most of the simulation time.

**Fig. 7:**
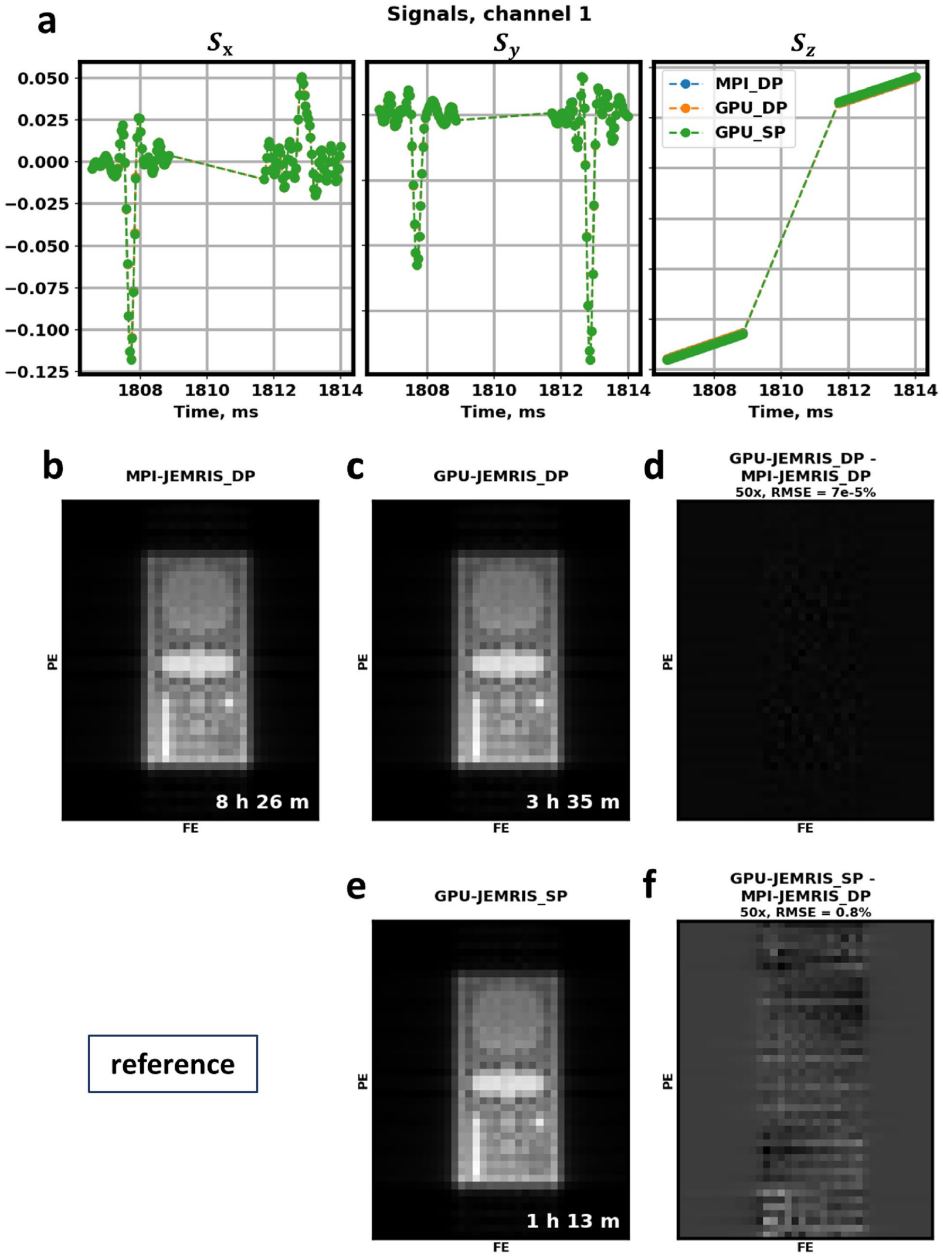
Double and single precision GPU simulations compared to double precision MPI for a geometrical phantom with 2,250,000 spins and a 2D TSE sequence. (**a**) Received signals for the first two spin echoes in coil channel 1. GPU and CPU results closely match with the total signal NRMSE of $0.11\%$ (double precision) and 0.10% (single precision). IFFT-reconstructed images from receive coil channel 1: (**b**) double precision MPI, (**c**) double precision GPU, and (**e**) single precision GPU. Difference images: (**d**) double precision GPU vs. MPI (NRMSE $=7e^{-5}\%$), (**f**) single precision GPU vs. MPI (NRMSE $=0.8\%$). Computation times reduced from 8.5 h (double precision MPI) to 3.5 h (double precision GPU) and 1.25 h (single precision GPU)

**Fig. 8:**
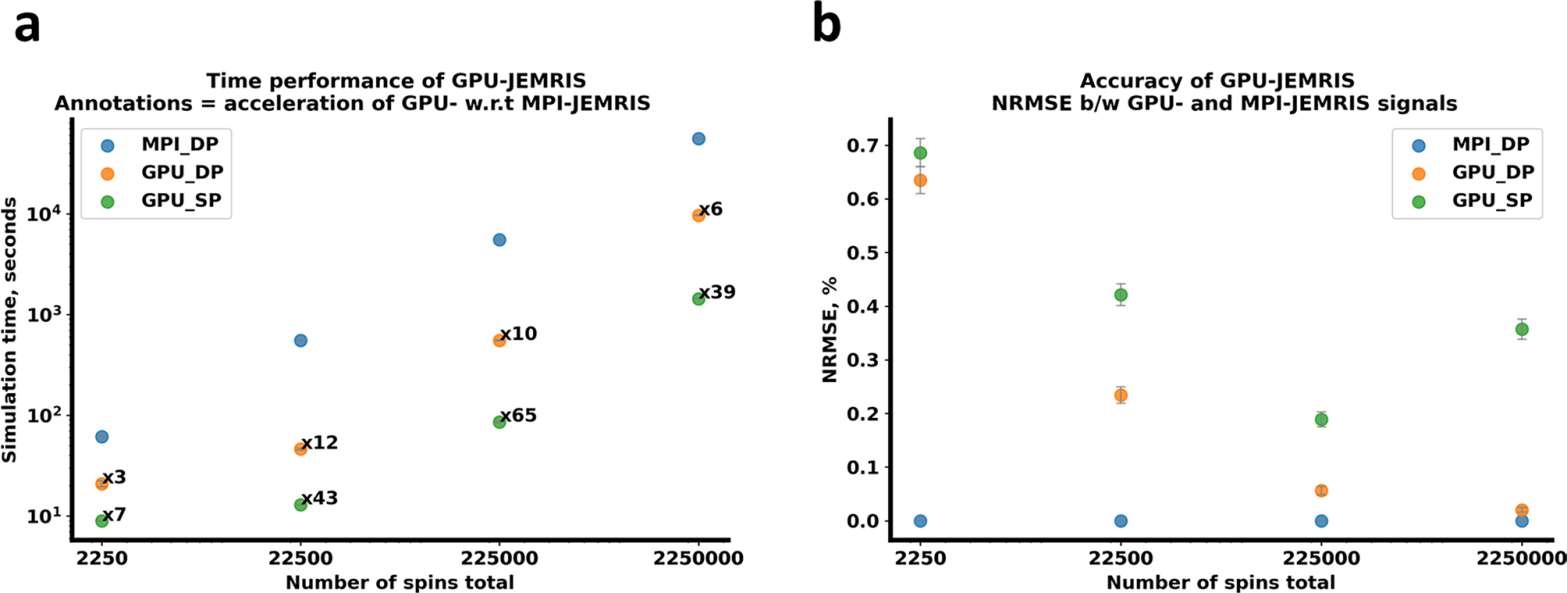
Acceleration and accuracy analysis of GPU simulations with increasing number of spins in a geometrical phantom. (**a**) Simulation times as a function of the total number of spins (in a logarithmic scale) for double precision MPI ($MPI\_DP$), double and single precision GPU ($GPU\_DP$ and $GPU\_SP$) simulations. Speed-up factors of 3–12 were achieved for double precision and 7–65 for single precision GPU simulations vs. double precision MPI simulations. (**b**) K-space signal normalized root-mean-square error (NRMSE) for double and single precision GPU simulations compared to the MPI reference. The NRMSE was below $0.08\%$ for double precision and between 0.2–0.8% for single precision GPU simulations

### Accuracy results

The accuracy of simulations with the GPU model was tested on the three artifact examples from the original JEMRIS paper [18]. Figure 6 shows banding artifacts in balanced steady-state free precession (bSSFP) acquisition simulated by double and single precision GPU-JEMRIS versus simulations using double precision MPI-JEMRIS. The IFFT-reconstructed images appear similar from double precision GPU-JEMRIS simulations (Fig. 6b) and MPI-JEMRIS (Fig. 6a) and have small random pixel intensity differences visible in Fig. 6c. The double-precision simulations on GPU took 71 s, compared to 116 s on CPU. At the same time, the single-precision bSSFP simulation on GPU (Fig. 6d) took 18 s and shows comparable image quality the double precision model on device (Fig. 6e) with the NRMSE less than $1\%$.

Simulations of chemical shift artifacts in EPI and of long refocusing pulse artifacts in TSE are presented in Figs. Suppl. S2 and Suppl. S3 respectively. In both examples, simulations with GPU-JEMRIS in both double and single precision exhibited image quality comparable to that of simulations with double precision MPI-JEMRIS resulting in k-space NRMSE values below $1\%$ for all four simulations. Accelerations factors of 8 and 40 were respectively achieved for double and single precision GPU simulations of the chemical shift artifacts, and x3 acceleration—for single precision GPU computations of the TSE acquisition. The double precision GPU simulation of the TSE acquisition was 1.5 times slower compared to the MPI simulations Suppl. S3b.

### Speed results

Figure 7 presents the spin echo signals and reconstructed images for the first coil channel in simulation with 2.25 million spins from Fig. 4. All $S_x$, $S_y$ and $S_z$ components in the double and single precision GPU simulations exhibited subtle deviations when compared to the double precision MPI simulations (Fig. 7a). Overall, the signal NRMSE were correspondingly $7 \times 10^{-5}\;\%$ and 0.8% for double and single precision GPU simulations, respectively, compared to the double-precision MPI simulations. The reconstructed images for the device computations were in agreement with the computations on CPU (Fig. 7b, c, e), showing subtle differences in the phase-encoding direction with single precision GPU simulations (Fig. 7d, f).

Figure 8 and Table 2 summarize the accuracy and performance results for double and single precision GPU models compared to MPI-CPU simulations for the geometrical phantom with an increasing number of spins. The simulation time for the MPI-JEMRIS scaled linearly with the total number of spins. However, the simulation time curves for both versions of the GPU simulator have a sigmoid-like shape. The double and single precision GPU simulations exhibited acceleration factors of 3–12 and 7–65, respectively (Fig. 8a). The ratio between the double and single precision computational times on GPU was 4x on average.

**Table 2:** Simulation runtime mean and standard deviation in [seconds] for MPI-parallelized JEMRIS (MPI-JEMRIS) with 24 processes, as well as for GPU-accelerated JEMRIS in double precision (GPU-JEMRIS, DP) and single precision (GPU-JEMRIS, SP). The benchmarking experiments were performed using a Turbo Spin Echo (TSE) sequence exported from a clinical MRI scanner and 4-channel coil sensitivities, with varying numbers of spins. The last two columns report the acceleration factors achieved with GPU-JEMRIS in double and single precision, respectively, compared to MPI-JEMRIS.

Number of spins	MPI-JEMRIS DP	GPU-JEMRIS DP	GPU-JEMRIS SP	Acceleration, GPU_DP	Acceleration, GPU_SP
2250	$61.3 \pm 0.6$	$21.0 \pm 1.0$	$9.0 \pm 0.5$	3	7
22,500	$555.3 \pm 9.0$	$46.3 \pm 0.6$	$13.0 \pm 0.3$	12	43
225,000	$5555.0 \pm 4.4$	$554.7 \pm 5.0$	$86.0 \pm 1.3$	10	65
2,250,000	$55626.0 \pm 3.5$	$9647.7 \pm 2.1$	$1438.0 \pm 1.1$	6	39

In terms of accuracy, the k-space NRMSE values between the GPU and MPI results were respectively 0.007–0.073% and 0.2–0.8%, being higher for the single precision computations and increasing with the number of spins (Fig. 8b).

Simulation times mean and standard deviation for KomaMRI.jl and JEMRIS were compared across five experiments for both CPU and GPU implementations, as shown in Table 3 and Table 4, respectively. Under matched conditions, KomaMRI.jl demonstrated a 50–200 times speedup on CPU and a 6–70 times speedup on GPU compared to JEMRIS. The simulation results from two frameworks had NRMSE less than $1\%$ for the studied scenarios.

**Table 3:** Simulation runtime mean and standard deviation in [seconds] with JEMRIS and single-threaded KomaMRI.jl on CPU using Intel Xeon W-2145 in the five experiments from [14]

Experiment	JEMRIS	KomaMRI.jl CPU, DP	ratio	MPI-JEMRIS, np = 8	KomaMRI.jl CPU, DP, 8 threads
a. EPI of a 1D column	4.26 ± 0.19	0.06 ± 0.002	71	0.85 ± 0.03	0.08 ± 0.02
b. EPI of circles	135.31 ± 2.90	1.60 ± 0.02	85	19.46 ± 1.29	0.08 ± 0.02
c. EPI of brain	383.33 ± 0.47	6.31 ± 0.01	61	15.00 ± 0.00	1.18 ± 0.06
d. EPI of brain with motion	451.12 ± 5.74	8.33 ± 0.001	54	64.88 ± 0.71	2.06 ± 0.05
e. Spiral GRE in brain	916.29 ± 3.93	5.01 ± 0.28	183	153.31 ± 1.50	0.99 ± 0.04

The last two columns show the parallelized CPU simulations with MPI-JEMRIS and multi-threaded KomaMRI.jl respectively, although these runtimes cannot be compared directly. Across the five experiments, KomaMRI.jl performed the simulations 50–200 times faster

**Table 4:** Simulation runtime mean and standard deviation in [seconds] with JEMRIS and KomaMRI.jl on GPU using double and single precision in the five scenarios presented in [14]

Experiment	GPU-JEMRIS, DP	KomaMRI.jl GPU, DP	ratio DP	GPU-JEMRIS, SP	KomaMRI.jl GPU, SP	ratio SP
a. EPI of a 1D Column	1.46 ± 0.05	0.02 ± 0.003	73	0.96 ± 0.03	0.02 ± 0.003	48
b. EPI of circles	2.01 ± 0.07	0.11 ± 0.006	18	1.28 ± 0.04	0.10 ± 0.004	13
c. EPI of brain	2.63 ± 0.05	0.30 ± 0.004	9	1.39 ± 0.02	0.21 ± 0.005	7
d. EPI of brain with motion	2.26 ± 0.09	0.39 ± 0.002	6	1.32 ± 0.05	0.26 ± 0.001	5
e. Spiral GRE in brain	7.71 ± 0.35	0.31 ± 0.003	25	1.38 ± 0.22	0.23 ± 0.004	6

Experiments were run on NVIDIA A6000. In these five experiments, KomaMRI.jl performed GPU simulations 6–70 times faster

### Results of quantitative imaging in the presence of motion

**Fig. 9:**
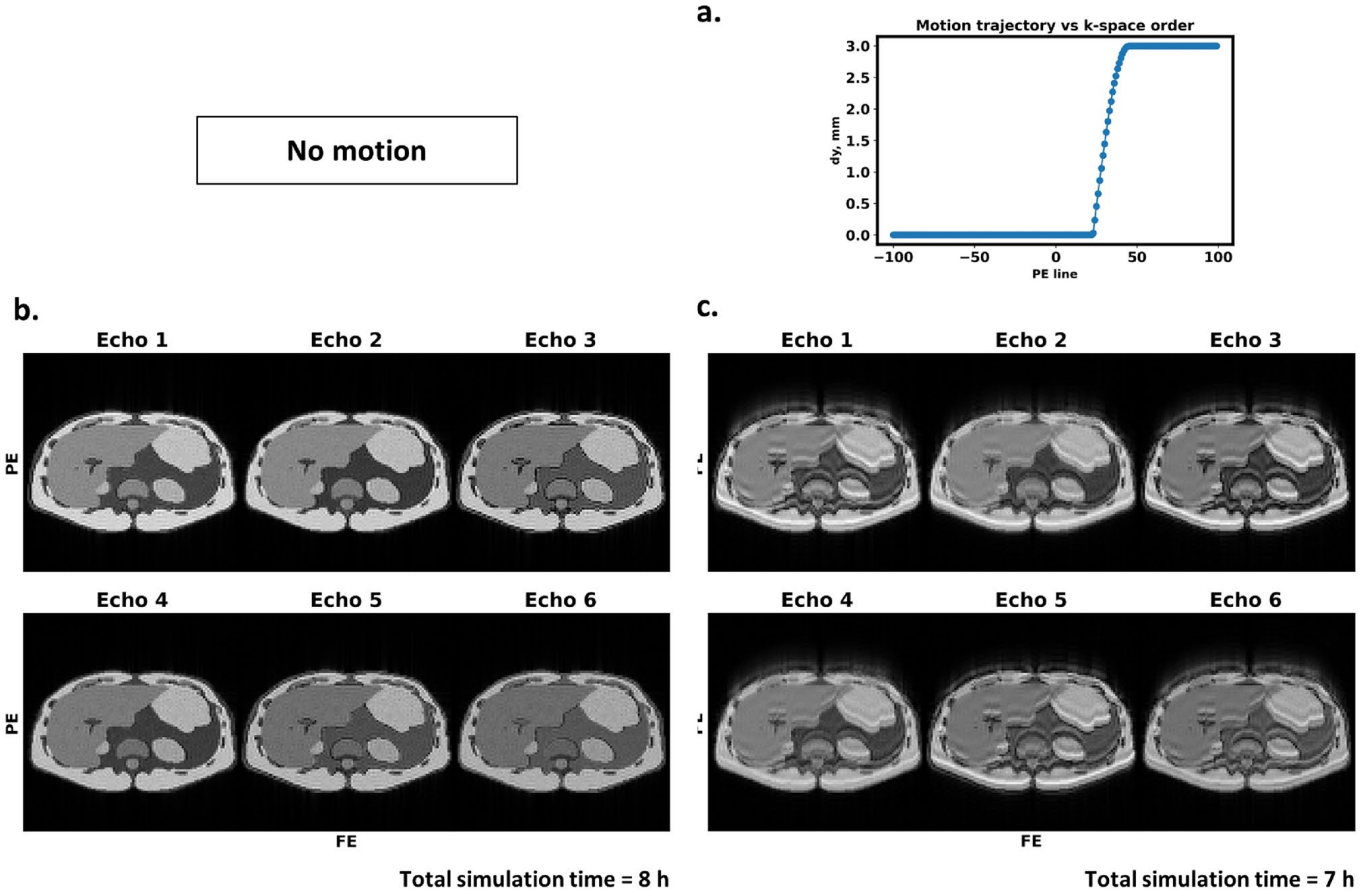
Multi-echo Dixon sequence simulations of a multi-species liver phantom in the presence of motion. (**a**) Motion trajectory plotted against the acquired phase encoding line. Translations of amplitude 3 pixels in the anterior-posterior direction were applied in the second half of data acquisition. (**b**) Magnitude images from the static phantom simulation present homogeneous and well-defined structures, while (**c**) images from the moving phantom simulations contain blurring and ghosting artifacts with similar patterns but varying severity across the echoes

Bloch simulations of the multi-species abdominal phantom and respiratory-like motion during the six-echo multi-echo gradient echo sequence enable the isolation of motion artifacts from other system and reconstruction imperfections. Figure 9b shows the $T_1$-weighted simulated images at the six echo times in the absence of motion. Homogeneous intensity within the tissues, sharp edges, as well as variations in the appearance of the water-fat signals across echo times could be noticed. Ghosting and blurring artifacts appear in Fig. 9c in the simulation results with the motion trajectory shown in Fig. 9a. Each simulation took 7–8 h on device with the single precision model and was not feasible with our CPU setup.

**Fig. 10:**
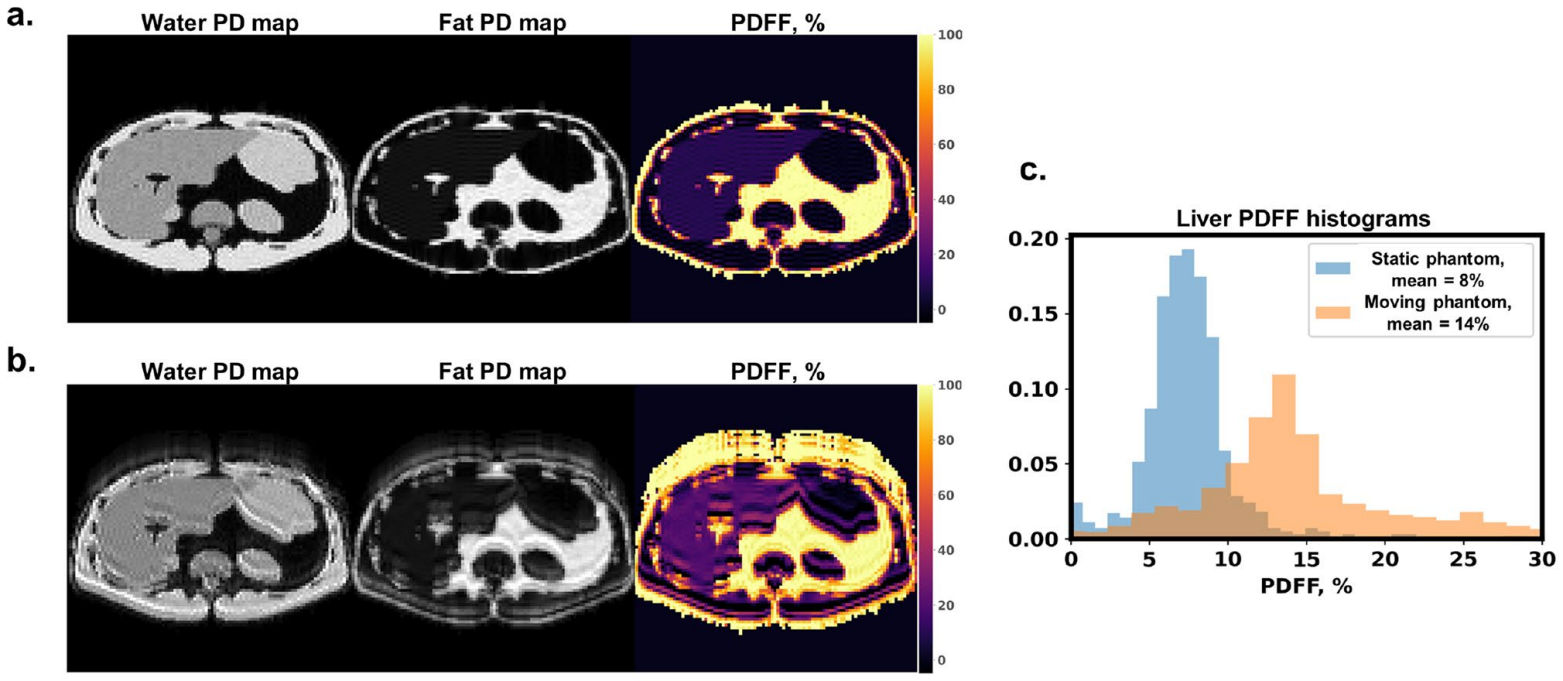
Fat fraction quantification errors induced by motion. Separated water and fat proton density, as well as PDFF maps for simulations with (**a**) static phantom and (**b**) moving phantom. (**c**) Histogram of estimated PDFF values in the liver region for static and moving phantom simulations. The ground truth liver PDFF was 10%. For the static phantom, the distribution of PDFF is narrow with mean value at $8\%$ and standard deviation $2\%$. In the presence of motion, the estimated PDFF values are distributed with mean $14\%$ and standard deviation $3\%$

Further, running the chemical shift encoding-based water-fat separation method allowed quantifying the effects of motion on liver PDFF values from simulations. Figure 10b for dynamic phantom simulations shows water-fat swaps having a ghosting pattern similar to the one in echo images (Fig. 9c). Further, liver PDFF value histograms from static and moving phantom simulations are presented in Fig. 10c. For the static case, the PDFF values were narrowly distributed with the mean at 8%. In the presence of motion, the distribution of PDFF values was broadened with the mean shifted to 14%.

## Discussion

The developed GPU-JEMRIS framework demonstrated speedup factors of 3–12 and 7–65 over MPI-JEMRIS using double and single precision ODE solvers, respectively, in a range of Bloch simulations. Benchmarking double versus single precision GPU simulations showed significant performance gains for single precision, despite the brute-force data precision conversion. These results fall within the expected GPU acceleration ranges observed in SUNDIALS forums and in [25], the latter reports speedups of roughly 2–10 on NVIDIA V100 GPUs, compared to multi-core CPU computations, for problems with 0.1 million to 1 million spatial grid points. With the current GPU-JEMRIS implementation, computational speedup over the CPU simulator is from parallelization along the spins dimension. Device memory allocation, data transfer and kernel launches have execution overheads, consequently, mid-to-large-sized problems are more likely to yield performance benefits that offset these costs when sufficient compute power is available.

Despite the availability of numerous GPU-based Bloch simulators [10, 11, 13–15, 17, 34], this work chose to extend the JEMRIS simulator, which has advantages of being general, extensible and having minimal physics modelling assumptions. JEMRIS handles numerical phantoms and multi-channel coils which can be generated in the GUI or exported from any programming language simplifying its use. The simulator’s key strength, however, is its sequence infrastructure—with basic sequences implemented and the possibility to extract arbitrary sequences and pulses from clinical scanners as it was done in [35]. This work focused on macro-level modifications for the GPU extension of JEMRIS, including efficient data transfer, minimizing kernel launches, optimizing grid shape, enabling asynchronous operations, and using single precision arithmetic. However, kernel-level optimizations (e.g., shared memory, dynamic or warp-level tuning) were not applied. Since $\sim 80\%$ of runtime is spent within CVode and CUB libraries, performance largely depends on their optimizations.

Benchmarking against KomaMRI.jl showed that GPU-JEMRIS ran 5–20 times slower. Benchmark timings excluded KomaMRI.jl’s JIT compilation time, which may be a relatively larger proportion of runtime for small problems but is insignificant for larger problems. This benchmark highlights how matrix-based Bloch simulators offer efficiency and simplicity compared to ODE-based methods. Among these, the most commonly employed symmetric and asymmetric operator-splitting methods, achieving second- and first-order convergence, respectively, with respect to the chosen time step. However, fixed time discretization can become practically limiting, especially for sequences with rapidly varying components that demand small time steps to maintain accuracy [36]. Applying such small steps uniformly across the entire simulation substantially increases computational costs. In this context, adaptive ODE-based simulators provide a reliable reference for validating accuracy, offering higher theoretical convergence (third order or beyond) without requiring manual adjustment of time steps for capturing rapid dynamics, such as short RF pulses, strong gradients or short relaxation properties. Leveraging GPU-accelerated ODE solvers could help mitigate the increased per-step computational burden, which inherently depends on the specified error tolerance.

Accuracy metrics showed that double precision GPU-JEMRIS produced signals with NRMSE below 0.1%, closely matching the original CPU simulator, with deviations close to compute precision as the number of spins increased. Single precision GPU-JEMRIS also demonstrated good agreement, with NRMSE remaining below 1% in the studied cases (Fig. 8). The benchmarking also indicated, in a time-matched experiment, GPU acceleration alone could enable simulations with $\sim 3\times$ more spins, whereas combining GPU with single precision could allow for $\sim 30\times$ more spins compared to CPU-based double precision. This increased spin count might be useful for studying statistical effects with ODE-based Bloch simulations. To highlight the importance of discretization tuning with Bloch simulations, we described an experiment with tuning the number of spins per voxel for gradient spoiling sequence in Suppl. Fig. S4. Similar tests defining the sample resolution at which simulation results converge, were performed for each problem presented.

Finally, limitations of the developed GPU-JEMRIS currently include missing diffusion, chemical exchange, parallel transmission, and magnetization transfer functionalities, which are available in the CPU version. The current single-GPU implementation does not include memory management strategies for problem sizes exceeding the GPU’s available memory. The extension is presently limited to devices with CUDA support and the Sundials package version $>4.0$, although adapting it to HIP would be feasible with little effort, as HIP is supported in CVode. An MPI-GPU extension using the existing *pjemris* backbone could be also considered to further accelerate Bloch simulations with JEMRIS.

## Conclusions

A GPU-accelerated extension for JEMRIS was developed, demonstrating speed up factors of 3–12 and 7–65 over MPI-simulator using double and single precision ODE solvers, respectively, for the investigated scenarios. All experiments showed k-space NRMSE below $1\%$ when compared to the CPU reference. GPU-JEMRIS enables Bloch simulations by solving ODEs directly on a GPU, which could provide new insights into more realistic, large-scale spin pool problems—including those involving larger phantoms, multiple species, and motion.

## Supplementary Information

Below is the link to the electronic supplementary material.

Supplementary file 1 (pdf 2384 KB)

## Data Availability

The code for the simulation framework and described experiments is already publicly available under https://github.com/BMRRgroup/gpu-jemris.
