# Supplementary material for: Gpu-accelerated JEMRIS for extensive MRI simulations
Source: MAGMA. 2025 Sep 4;38(4):679–94. doi: 10.1007/s10334-025-01281-z (PMC12443918; doi:10.1007/s10334-025-01281-z)
Supplement: Supplementary file 1 — (pdf 2384 KB) [file 10334_2025_1281_MOESM1_ESM.pdf]

8 Supplementary Figures

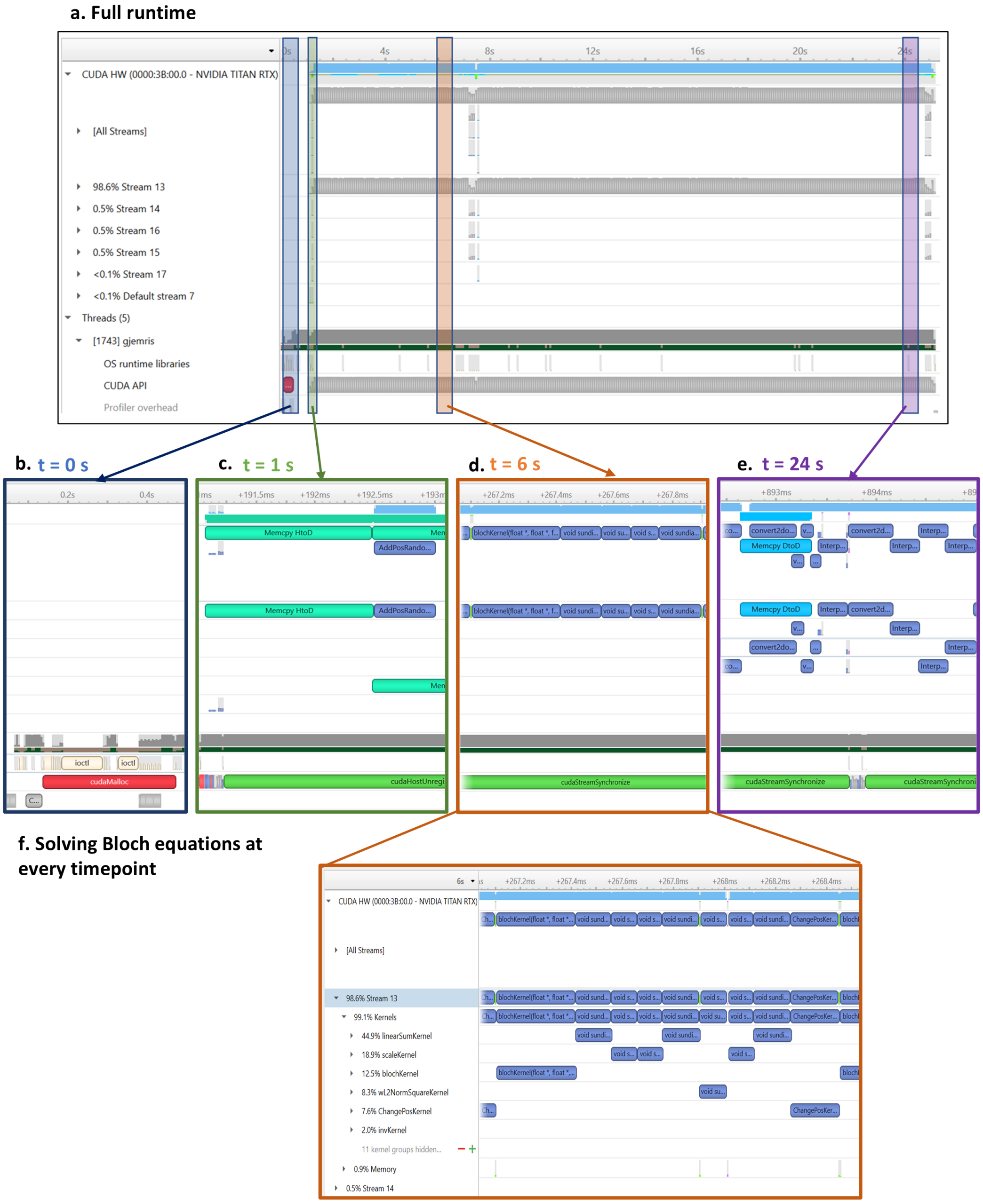

Fig. S1 A screenshot from NVIDIA Nsight Systems profiler visualizing GPU-JEMRIS simulations of 2 spin-echoes with a geometrical phantom (2.25 mln spins) and 4-channel receiver coils. a) Full simulation profile: main CUDA stream handles most computations; three additional streams enable parallel host-device transfers and multi-coil reception, ensuring high GPU occupancy throughout runtime. b) Zoom-in at $t = 0\,s$ highlights CUDA memory allocation occurring only at simulation startup, with no further allocations visible in (a). c) Zoom-in at $t = 1\,s$ shows host-to-device numerical sample transfers partially overlapping initialization kernels. d) Zoom-in at $t = 6\,s$ captures GPU computations solving Bloch equations at each timepoint. e) Zoom-in at $t = 24\,s$ depicts signal reception: magnetization vectors multiplied by coil sensitivity maps, converted to double precision, and reduced on GPU; each coil uses a separate CUDA stream, transferring signals simultaneously to host. f) Extended view of (d) provides details on frequently executed Bloch equation integration kernels.

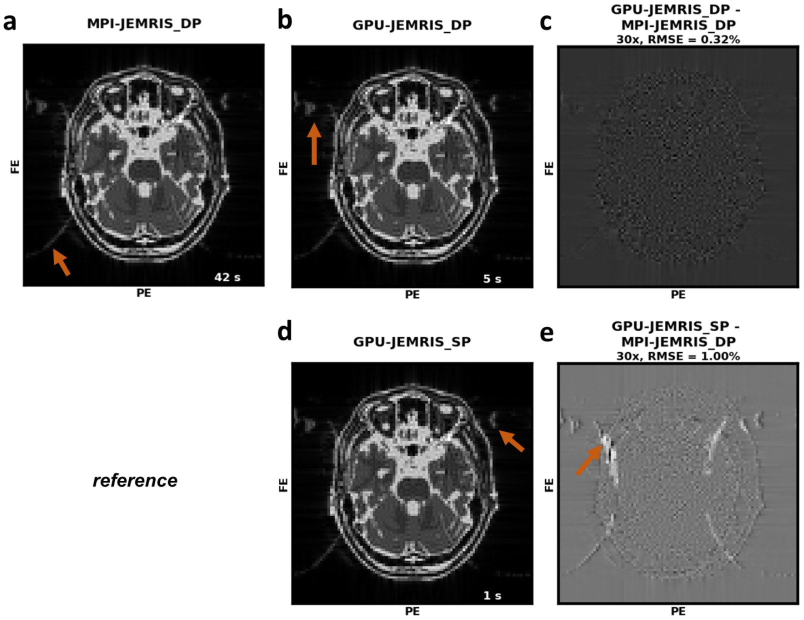

Fig. S2 Reproducing simulations of common MRI artifacts from [18]: chemical shift artifacts in EPI acquisition. The numerical brain phantom contains fat tissue which causes wrap-around artifacts on the IFFT-reconstructed image. IFT-reconstructed images from a) double precision MPI-JEMRIS, b) double precision GPU-JEMRIS, and d) single precision GPU-JEMRIS, with corresponding difference maps shown in c) and e). Simulation runtimes were 42 s (MPI-JEMRIS), 5 s (double precision GPU-JEMRIS), and 1 s (single precision GPU-JEMRIS). Total NRMSE remained below 1% for both GPU simulations compared to CPU simulations.

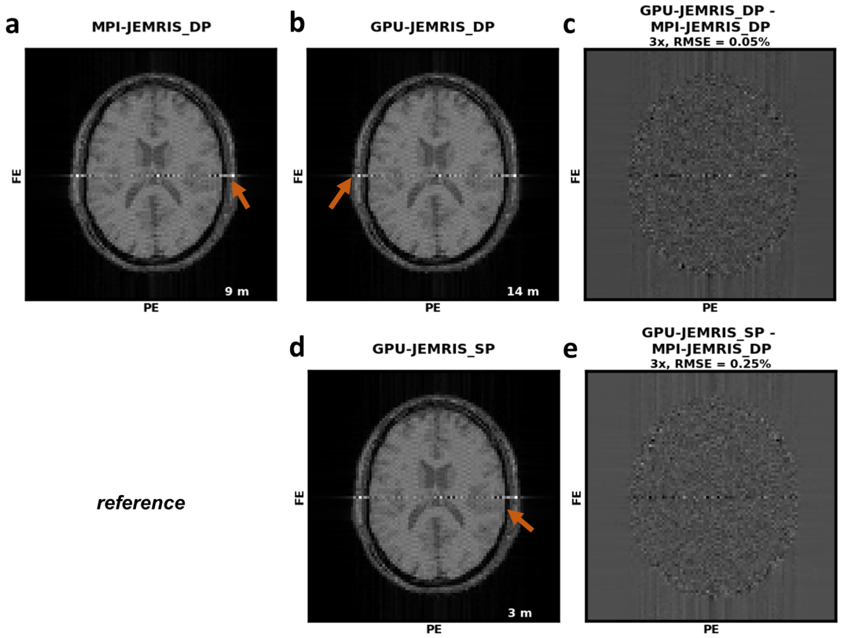

Fig. S3 Reproducing the simulations of common MRI artifacts from [18]: artifacts from a long refocusing pulse in a spin echo sequence. IFT-reconstructed images from a) double precision MPI-JEMRIS, b) double precision GPU-JEMRIS, and d) single precision GPU-JEMRIS, with corresponding difference maps shown in c) and e). Simulation runtimes were 9 mins (MPI-JEMRIS), 14 mins (double precision GPU-JEMRIS), and 3 mins (single precision GPU-JEMRIS). Total NRMSE remained below 0.25% for both GPU simulations compared to CPU simulations.

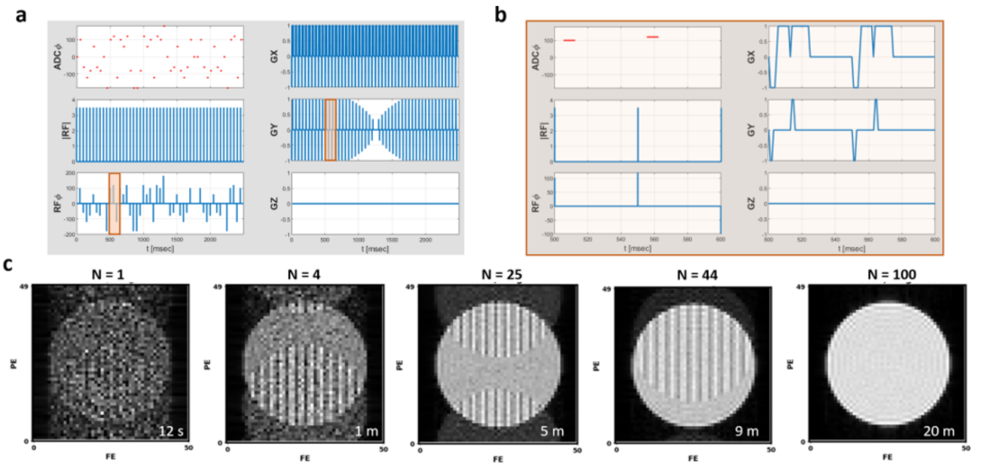

Fig. S4 Tuning the number of spins per voxel necessary for gradient spoiling simulations. a) Six-echo GRE sequence used in simulations, with a zoomed-in view in b) highlighting gradient spoilers along the frequency-encoding direction (G_x). c) Simulated images obtained with increasing spins per voxel. Only at 100 spins per voxel does the image show a homogeneous signal across the disc. Computation times, shown at the bottom-right of each image, range from 12 seconds (1 spin/voxel) to 20 minutes (100 spins/voxel).
